# Sap Flow Sensors: Construction, Quality Control and Comparison

**DOI:** 10.3390/s120100954

**Published:** 2012-01-16

**Authors:** Tyler W. Davis, Chen-Min Kuo, Xu Liang, Pao-Shan Yu

**Affiliations:** 1 Department of Civil and Environmental Engineering, University of Pittsburgh, 3700 O’Hara Street 941 Benedum Engineering Hall, Pittsburgh, PA 15261, USA; E-Mails: twd2@pitt.edu (T.D.); jemkuo@mail.ncku.edu.tw (C.-M.K.); 2 Department of Hydraulic and Ocean Engineering, National Cheng Kung University, Tainan City, Taiwan; E-Mail: yups@mail.ncku.edu.tw

**Keywords:** environmental sensors, heat ratio method, thermal dissipation method, sap flow

## Abstract

This work provides a design for two types of sensors, based on the thermal dissipation and heat ratio methods of sap flow calculation, for moderate to large scale deployments for the purpose of monitoring tree transpiration. These designs include a procedure for making these sensors, a quality control method for the final products, and a complete list of components with vendors and pricing information. Both sensor designs were field tested alongside a commercial sap flow sensor to assess their performance and show the importance for quality controlling the sensor outputs. Results show that for roughly 2% of the cost of commercial sensors, self-made sap flow sensors can provide acceptable estimates of the sap flow measurements compared to the commercial sensors.

## 1. Introduction

The heat ratio [1], heat balance [2], and thermal dissipation [3] sap flow methods make up the three main sensor types that are used today for estimating plant transpiration. With respect to certain tree sizes (>25 cm diameter), only the heat ratio method (HRM) and the thermal dissipation method (TDM) are applicable [4]. Both of these methods use cylindrical thermocouple and heater probes inserted into the tree xylem. While the installation and methodology of using these sensors are different, the fundamental mechanics and operation of the probes are similar. Reference [4] suggests that a combination of both pulsed and thermal dissipation sap flow measurement methods would be the most cost effective solution for long-term studies. In addition, the high cost associated with commercial brand sensors of the same types, *i.e.*, TDM and HRM, diminishes the ability for researchers to make large-scale deployments. Therefore, this study describes the design of both a HRM and TDM sap flow sensor utilizing inexpensive parts for the purpose of estimating tree sap flow.

To accompany the design of these two sensors, a quality control checking method is also presented. Due to the inconsistencies that may arise when producing handmade sap flow sensors, it is important to calibrate each probe’s output to a standard measure. This paper presents a simple method to do so and the results from calibrating 24 temperature and heater probes’ output (measured in voltage) to a standardized temperature (measured in degrees). This accounts for the variations in individual sensors by applying a linear conversion equation to the sensor probes’ output.

To demonstrate the sensor’s performance, results from a field study using both HRM and TDM handmade sensors are also presented. For further comparison, a commercial sap flow sensor, Dynamax Inc.’s TDP30, was field tested alongside the handmade sensors. Five consecutive days’ results from all three sensors are presented. Simultaneous measurements from three different sensor types measuring at three different locations in the same tree cannot present the same quantitative results due to heterogeneities present in the thermal properties of the tree’s sap-conducting wood [5]. Therefore, only a qualitative comparison is made.

The remainder of this paper is organized as follows: Section 2 overviews the background information regarding the two sap flow sensor methodologies and their existing sensor designs. This includes the motivation for building self-made sensors as opposed to purchasing commercially. Section 3 covers the methods for building both sap flow sensor types and the materials and pricing for the sensor parts. The results and conclusions based on the field tests of both sensors are given in Section 4 with a comparison against a commercially purchased TDM sensor. A discussion is provided in Section 5.

## 2. Background and Motivation

### 2.1. Overview of Sap Flow Methodologies

The TDM was first introduced by [3]. This method is based on the assumption that the heat input by the sensor under steady sap flow conditions is equal to the heat dissipation (via convection and conduction) along the interface between the sensor and the tree when the sensor and the tree are in thermal equilibrium. Daily fluctuations in the heat dissipated from the sensor probe are compared to the unheated temperature of the tree sap and wood. To measure the heated and reference temperatures, two probes, vertically aligned, are inserted radially into the sapwood of a tree. The downstream probe consists of a coiled metal wire, which supplies the heat via the Joule effect, and a thermo-junction, which measures the temperature via the Seebeck effect. A constant voltage is added to the coiled metal wire to power the heater while the thermo-junction measures the temperature of the heat source. The upstream probe consists of only a thermo-junction for measuring the unheated reference temperature at a fixed distance away from the heater probe. Daily temperature difference measurements are used to calculate the sap flux density from an empirical equation developed by [6]:

(1) $$Q_{s}=0.000119\cdot {\left(\frac{\Delta T_{o}-\Delta T}{\Delta T}\right)}^{1.231}$$

where Δ*T* is the temperature difference between the upper and lower probes (°C), Δ*T*_o_ is the maximum daily value of Δ*T* which corresponds to zero sap flow (°C), and *Q_s_* is the sap flux density (m^3^·m^−2^·s^−1^) [7]. Reference [8] outlines the various research papers that have reported calibrating the Granier-style sensor on a variety of tree species and porous media.

The HRM is an improved heat-pulse method developed by [1] in response to the limitations of the compensation heat-pulse method (CHPM) first introduced by [9] and later developed by [10] and [11]. This method uses the relative temperature increases following a heat pulse measured at equal distances upstream and downstream from the source to determine the convective velocity of the heat pulse which can then be compensated by the tree’s physical characteristics, *i.e.*, a weighted average of the stationary wood and flowing sap, to the sap velocity. In this method, three probes are inserted radially into the tree sapwood. The middle probe generates a heat pulse and the temperature changes are measured by the other two probes at locations equidistant upstream and downstream from the heater. The heat pulse velocity is calculated according to [10]:

(2) $$v_{h}=\frac{k}{x}\cdot \text{ln}\left(\frac{v_{1}}{v_{2}}\right)$$

where *v_h_* is the heat pulse velocity (mm·s^−1^) [7]; *k* is the thermal diffusivity of sapwood (mm^2^·s^−1^); *x* is the distance between the heat pulse probe and the temperature probes (mm); *v*_1_/*v*_2_ is the ratio of time-dependent temperature differences, *v*(*t*) = *T*(*t*) − *T*(0), measured at the downstream and upstream probes, respectively. The times, *t*, during which measurements are made should be between 60–100 s following the generation of the heat pulse when *v*_1_/*v*_2_ is effectively linear with respect to time [1]. The sap velocity is then calculated based on the methodology of [12]:

(3) $$v_{s}=\frac{\rho_{\mathrm{sm}}c_{\mathrm{sm}}}{\rho_{s}c_{s}}\cdot v_{h}$$

where *v_s_* is the sap velocity (mm·s^−1^) [7]; *ρ_sm_* and *c_sm_* are the density (kg·m^−3^) and specific heat (J·kg^−1^·°C^−1^) of the sap in a woody matrix; and *ρ_s_* and *c_s_* are the density (kg·m^−3^) and specific heat (J·kg^−1^·°C^−1^) of the sap. The heat pulse velocity in Equation (2) can be corrected for sensor misalignment and tree wounding effects which can affect the sap flow measurements [1]. Results of either Equation (1) or (3) can be multiplied by the cross-sectional area of conducting sapwood to convert sap flux density (*Q_s_*) or sap velocity (*v_s_*) to sap flow (*Q*).

### 2.2. Existing Measurement Technologies

The means for measuring sap flow have been designed and sensors for the HRM and TDM are commercially available. Reference [8] lists three major companies as distributers of the Granier-style sap flow sensors, including UP Gmbh (Germany), PlantSensors (Australia), and Dynamax (USA). Others include Ecomatik (Germany) and ICT International (Australia). ICT International is the only known supplier of a sensor specifically for the HRM. These sensors, however, are expensive and often times packaged with proprietary data collection equipment and software which can also be expensive and limited in functionality.

The fundamental principles of these sap flow sensors are based on the thermo-electric and Joule heating laws. Using these principles, temperature and heater probes can be created for the HRM and TDM similar to those described in their respective works or those sold by commercial sap flow sensor retailers. In their design of a variable length TDM sensor, Reference [13] states the inexpensive nature and simplicity of manufacturing and using self-made sap flow sensors. It is the intention of this work to exemplify this with simple sensor designs, quality control measures and an example application comparing the self-made sensors to their commercial counterparts.

Self-made sap flow sensors have been presented in numerous other works, e.g., [13–16], but most fail in describing at least one of the following: the process in which the sensors were made, the materials used, or the quality assurance of each sensor. This work details each of these key points.

### 2.3. Benefits of Using Self-Made Sap Flow Sensors

There are many benefits to making sap flow sensors rather than purchasing them commercially. These benefits are mainly due to the selection and pricing of materials used for the sensor building. The time and experience necessary to make the sensors may be offset as an educational cost.

The parts for making the probes comprised in the sensor design for both the HRM and TDM sap flow sensors are the same, only in different quantities. Therefore, researchers can take advantage of using both sensor designs in their study which has been suggested for improving the quality of measurements [4]. Purchasing the sensor parts also allows for custom fitting the sensors to the specific application. This can include the probe length (to account for various sapwood depths), probe spacing (especially for the TDM where distance between the heater and reference temperature probes may affect results [8]), and wire connection type (to work with various data logger or collection systems).

The price of self-made sensors, when considering both time and materials, is much less than their commercial counterparts for large deployments. For small deployments, however, the trade-off between the quality and price between self-made and commercial sensors may not be as justifiable. However, large deployments allow researchers to install a greater number of sensors over larger areas and species types. This can help reduce errors in transpiration estimation made by single point measurements. It is well known that the TDM is prone to errors due to variations of sap flow along the sensor length, improper probe placement, disruption to conducting cells during installation, improper probe spacing and temperature gradients within the sap wood [17]. To help accommodate for this, it is recommended that multiple sensor types be deployed together. Because both the TDM and HRM sap flow sensors share the same parts, they can be built and deployed together for a dual perspective of sap flow.

Time and effort are necessary to assemble sap flow sensors. The experience of the worker will affect the quality of the sap flow sensors produced. The time spent on sensor production may delay deployment, however, the experience gained from making sensors is valuable. The practice of building sap flow sensors has proven to be a successful hands-on training tool and an effective learning experience for undergraduate student education. Making sap flow sensors, while time consuming and repetitive, does not require specialized skill sets outside an undergraduate student’s capabilities. Therefore by providing this as an opportunity for undergraduate work, the additional cost of making the sensors is avoided.

## 3. Methods and Materials

This section presents two sensor designs for the TDM and HRM sap flow calculation methods. The designs for building the sap flow sensors are based on [18,19]. The principal components of these two sensors have been reduced to three probe designs. From these three probes, both the TDM and HRM sap flow sensors can be constructed. The TDM and HRM sap flow sensor designs both utilize a temperature probe (for the reference temperature in the TDM and the equidistant temperature measurements upstream and downstream in the HRM). The difference between these two sap flow sensors are their heater probes. The TDM utilizes a constant heater probe, consisting of both a heater and a temperature measurement, while the HRM utilizes a heat pulse probe, consisting of only a heater.

To supply the constant heat required by the TDM, a design for a voltage regulator is necessary and is included in this paper. Unlike the HRM, which uses a short but high power heat pulse, the TDM delivers a steady and constant power output which in this design is controlled via a voltage regulator.

To control the quality of these probes, their measurements (measured in mV) are calibrated to a standardized thermocouple (measured in °C). The testing procedure and results from two tests are presented below.

### 3.1. Construction and Quality Control

#### 3.1.1. Temperature Probe

**Figure 1.**
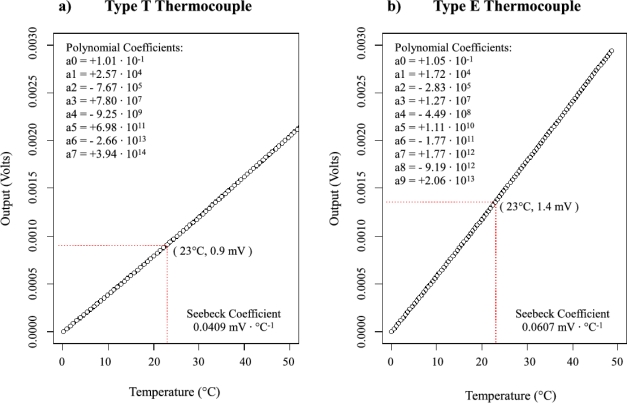
(**a**) Type T thermocouple voltage to temperature conversion plot. (**b**) Type E thermocouple voltage to temperature conversion plot. The polynomial voltage (V) to temperature (°C) conversion equation coefficients, Seebeck coefficients for the near linear temperature range shown and voltage responses at 23 °C are given for both thermocouple types.

The temperature probe used by both the HRM and TDM consists of a thermocouple junction, or thermo-junction, inside of a metal needle. Traditionally, a copper-constantan (Type T) thermocouple is used. In this design, the thermocouple type was changed to a chromium-constantan (Type E) to increase the voltage output. Figure 1 shows the comparison between Type T and Type E thermocouples. The thermocouples’ voltage response to a given temperature can be represented by a polynomial (of order seven and nine for Type T and E thermocouples, respectively) and have a nearly linear response curve in the temperature range expected for sap flow monitoring conditions. However, the Type E thermocouples produce over 50% higher voltage response than Type T for a given temperature (e.g., 1.4 mV compared to 0.9 mV at 23 °C for Type E and T thermocouples, respectively). Reference [20] shows that the TDM sap flux density can be directly calculated from the voltage measurements as opposed to the temperature, due to the cancellation of the conversion factor (Seebeck coefficient) in the numerator and denominator. Based on the ratio of time-dependent temperature differences in Equation (2), the same is also true for the HRM calculation. Therefore using a thermocouple with a higher Seebeck coefficient will not change the results in the calculation. Instead, for the same temperature measurement the Type E thermocouple will produce a higher voltage that can be more easily resolved by instrumentation.

**Figure 2.**
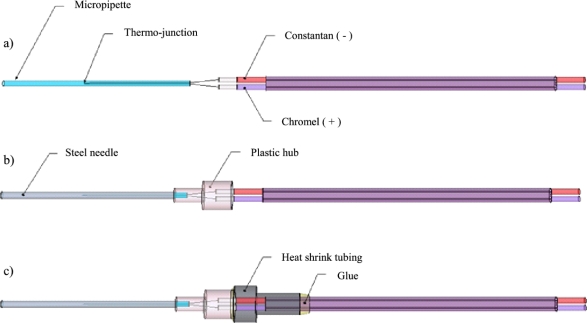
(**a**) Temperature probe schematic of thermo-junction location inside the micropipette and connection to Type E thermocouple extension wire (constantan/chromel). (**b**) Temperature probe schematic showing micropipette located inside the steel dispensing needle. (**c**) Temperature probe schematic of the heat shrink tubing and glue used to secure the thermocouple extension wire to the plastic hub of the needle.

The construction of the temperature probe begins with 36 gauge (0.127 mm diameter) chromel and constantan thermocouple wire. The wires are soldered to form a thermo-junction and inserted into a 41 mm long, 0.556 mm inner-diameter glass micropipette. The chromel wire is insulated with a PFA copolymer resin to prohibit short-circuits. The thermo-junction is glued into place inside the micropipette using a cyanoacrylate adhesive. The location of the thermo-junction in the micropipette determines the depth temperature measurements will be taken inside the tree. For this design, the thermo-junction is located at half the length of the micropipette. At this point, the thermocouple inside the micropipette can be tested using a multimeter to measure the resistance between the exposed leads to ensure the soldered joint was not damaged during installation. Depending on the quality of the soldered joint, resistance measurements typically are between 1–4 Ω. If resistance measurements are found to vary greatly from the expected, the thermocouple is discarded. Type T extension wires, 20 gauge (0.8128 mm diameter), are wrapped and soldered to their respective thin leads of thermocouple wire (Figure 2(a)). The connections are insulated with polytetrafluoroethylene (PTFE) tape. The micropipette is carefully inserted into an 18 gauge (0.965 mm inner-diameter) 38.1 mm long stainless steel dispensing needle (Figure 2(b)). It should be noted that the needle size was chosen to closely match that of the Dynamax TDP30 commercial sap flow sensor. The micropipette and needle size may be changed to match other designs. The tip of the steel needle is sealed with solder to restrict water from reaching the wiring. To hold the assembly together, heat shrink tubing is tightened around the needle hub and thermocouple extension wire. For additional water protection, the seam around the heat shrink tubing is glued (Figure 2(c)).

#### 3.1.2. Constant Heat Probe

**Figure 3.**
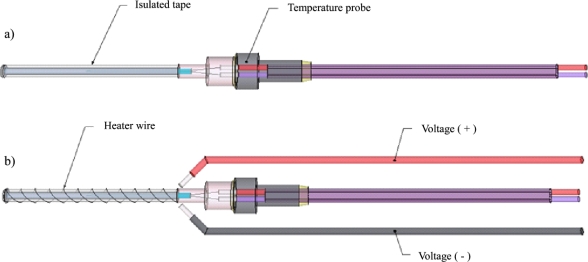
(**a**) TDM heater probe schematic of insulated tape covering the temperature probe’s steel dispensing needle. (**b**) TDM heater probe schematic of heater wire coiled around the insulated tape and connection to the positive and negative voltage extension wire.

The constant heat probe used by the TDM is a modification of the temperature probe described in Section 3.1.1. To provide the constant heat source required by the TDM, a heater wire is wrapped around the outside of the temperature probe’s needle. To reduce the potential for short circuiting the heater wire, the metal needle is first insulated with PTFE tape (see Figure 3(a)). Nickel-chromium wire (also known as Nichrome 60 and Chromel C) is then wrapped around the insulated needle (see Figure 3(b)). Note that the wire coil is not drawn to scale. At this point, the heater wire can be tested using a multimeter to measure the resistance between the two leads. If the resistance measured varies greatly from the expected, the wire is discarded. Positive and negative leads are attached to either end of the heater wire and are connected to a voltage regulator. For additional security against wire breakage, the heater leads can be tied to the thermocouple extension wire with electrician’s tape or by other means.

The 36 gauge (0.127 mm diameter) nickel-chromium wire has an approximate resistance of 88.6 Ω·m^−1^. Reference [3] calibrated the heater at 10 Ω and used a current of 0.141 A resulting in a 0.2 W powered probe. It is important to note that the calibration of this sensor depends on the heat field created by the probe (characterized by its size and shape) and the heating power used [8]. For this design, the heater resistance is set to approximately 45 Ω, therefore to maintain the 0.2 W power requirement of this sensor design, a 3.0 V power supply is required. It should be noted that the heater resistance and power voltage were decided based on the comparison with the Dynamax TDP30 sap flow sensor provided later. It is assumed that the heater resistance and power voltage can be adjusted to various combinations so long as the output power of 0.2 W is maintained.

The PTFE tape provides some support in holding the heater wire in place during installation. In Granier’s original design, the heater was placed into an aluminum sheath. The extent in which the aluminum sheath improves the thermal contact between the heater and the wood is unknown. Most installations today use wax or grease to improve thermal contact between the heater and wood in lieu of the aluminum sheath.

#### 3.1.3. Heat Pulse Probe

The HRM heat pulse probe, unlike that used by the TDM, does not measure temperature. Therefore the wire heater can be placed inside the probe needle where the thermo-junction was located in the temperature probe from Section 3.1.1. To reduce the potential for short circuiting the heater, the wire coil is placed inside a micropipette which is then inserted into the steel needle of the probe. The positive end of the heater wire is attached to heater extension wire, while the negative end of the heater wire is either also attached to the extension wire or soldered directly to the steel needle. In the latter case, negative extension wire is also soldered to the metal needle. The steel needle tip is sealed with solder to hold the negative end of the heater wire in place and prevent water intrusion. For quality assurance, the resistance of the heater probe between the positive and negative leads can be measured using a multimeter. If the resistance varies greatly from the expected, the heater probe is discarded.

**Figure 4.**
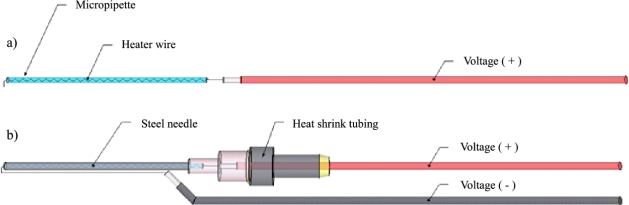
(**a**) HRM heater probe schematic of heater wire coil inside the micropipette connected to the positive voltage extension wire. (**b**) HRM heater probe schematic of micropipette located inside the steel dispensing needle, connection of the negative voltage extension wire to the heater wire, and heat shrink tubing and glue securing the positive voltage extension wire to the needle’s plastic hub.

The heater (Figure 4) is made of 36 gauge nickel-chromium (Nichrome 60/Chromel C) wire. To increase the temperature enough for detection in the upper and lower temperature probes during the short heat pulse, the resistance in this design was set at 20.1 Ω. Using a 12 V battery source, the probe delivers over 7.0 W of power.

#### 3.1.4. Voltage Regulator

**Figure 5.**
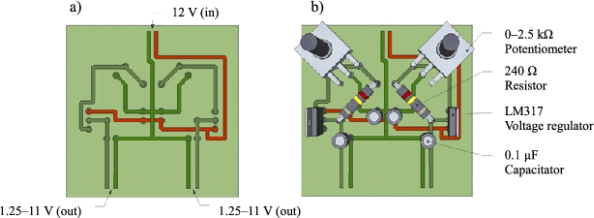
(**a**) Voltage dual regulator general wiring schematic. (**b**) Voltage dual regulator component locations and wiring schematic.

The TDM requires a steady constant power supply. Based on the TDM sensor design, the voltage that is required to maintain 0.2 W of power to the heater probe is approximately 3 V. To achieve this, a voltage regulator was designed which can reduce and split a single 12 V power source into two 1.25–11 V variable outputs. Each regulator requires only five components: an LM317 voltage regulator, a 240 Ω resistor, a 0–2.5 kΩ potentiometer, and two 0.1 μF capacitors. The dual design, given here, splits the 12 V input power in parallel to two regulators that are located on the same circuit board. This allows regulated voltage output to two separate constant heat probes. This design may be reduced to a single voltage regulator if desired. Figure 5 shows the layout of the components and a general wiring scheme.

#### 3.1.5. Sensor Output Calibration

Both the temperature probes and the TDM heater probes were tested for their response and accuracy to temperature measurements. This was to make certain that errors were not introduced into the probe temperature measurements due to variances in the craftsmanship. The testing consisted of comparing the temperature readings made by the sensor probes to a standardized thermo-junction. Each pair of measurements was recorded simultaneously in a series of three different water temperatures. The three water temperatures were: hot (∼40 °C), warm (∼25 °C), and cold (∼10 °C). A testing pattern of *cold* → *warm* → *hot* → *cold* → *hot* → *warm* was used such that each sensor probe was tested at each temperature twice.

Individual tests took three minutes (30 s for each temperature) and captured a gradual heating response, a rapid cooling response, a rapid heating response, and a gradual cooling response. The water temperature was measured and recorded every second using an Omega thermometer and temperature monitoring program (180 data points).

The TDM heater probes were also tested against the standardized thermocouple. Due to the insulation around the probe needle for the heater wire, each of the temperatures were tested for twice as long, resulting in a six minute total test time for each heater probe (360 data points).

### 3.2. Parts and Pricing

Table 1 shows the quantities for the various parts comprising the sap flow sensor probes and voltage regulator. The prices in Table 2 were taken from five commercial wholesale vendors for the year 2011. Table 2 shows the vendor and part numbers for all the materials listed in Table 1.

## 4. Results

Based on the three probe designs, the HRM and TDM sap flow sensors can be constructed. From the quantities given in Table 1 and the bulk pricings shown in Table 2, the temperature probe costs approximately $1.07, the constant heat probe costs approximately $1.17 and the heat pulse probe costs approximately $0.54. The HRM sap flow sensor, which is comprised of two temperature probes and one heat pulse probe, costs approximately $6.71 including three meters of extension wire. The TDM sap flow sensor, which is comprised of one reference temperature probe and one constant heater probe, costs approximately $6.27, which includes three meters of extension wire. The dual voltage regulator for the TDM costs approximately $6.68. The 2010 price guide from Dynamax lists the TDP30 sap velocity probes with 10 feet of extension wire at $330 each and the Dynamax Dual-Adjustable Voltage Regulator is priced at $340 each.

The TDM sensor designed for the field experiment was closely matched to the Dynamax TDP30 sensor. However, it should be noted that there are some minor differences between the TDM and Dynamax sensors. These included the thermo-junction type and the probe needle dimensions. The Dynamax TDP30 sap flow sensor uses Type T thermo-junctions and has a metal probe with a 30 mm length and a 1.27 mm outer diameter. The thermo-junction type was changed to Type E in the self-made design. As for the needle probe, the closest length available with a 1.27 mm outer diameter was 38.1 mm. Even though a longer needle is used for the self-made probes, the measurement length was found not to be a problem since the location at which the thermo-junction is positioned inside the probe can be placed to match that of the commercial sensor.

**Figure 6.**
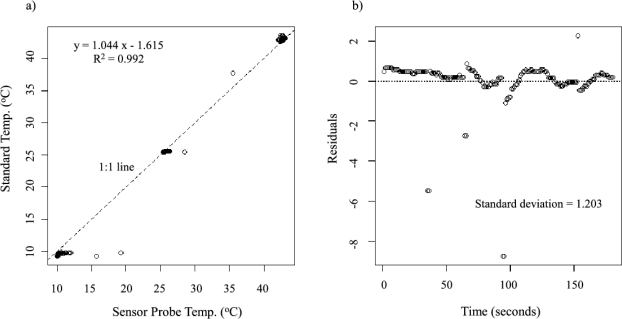
(**a**) Linear regression of a typical temperature probe’s output to the standard temperature. (**b**) Regression residuals from the temperature conversion equation and their standard deviation.

**Figure 7.**
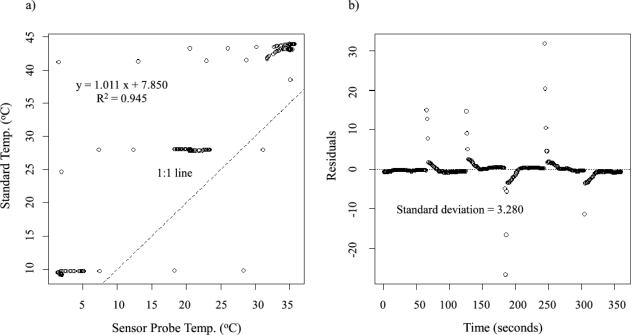
(**a**) Linear regression of a typical heater probe’s output to the standard temperature. (**b**) Regression residuals from the temperature conversion equation and their standard deviation.

Figures 6(a) and 7(a) show the calibration results from testing the sensor response of typical temperature and constant heat probes to a standard thermocouple temperature, respectively. Figures 6(b) and 7(b) show the residuals based on the regression between the probe output (measured in mV) and the standard temperature (measured in °C). The mean of the residuals of 24 temperature probes tested is 0.0276 °C, and the mean of the standard deviations of the residuals is 2.15 °C. For the constant heat probe, the insulation around the needle tends to cause underestimation of the actual temperatures (Figure 7(a)). Lag times for the rapid heating and cooling periods caused more outliers in the datasets, hence the longer test time. The mean of the residuals of 24 heater probes is 0.0315 °C, and the mean of the standard deviations of the residuals is 2.50 °C. In both cases, the mean of the residuals is close to zero and the mean of the standard deviations is small, indicating the stability and quality of the self-made temperature probes. However, the values are slightly larger for the constant heat probe compared to the self-made temperature probes.

The field measurements were made using both the self-made TDM and HRM sap flow sensors alongside the commercially available Dynamax TDP30 transpiration sensor. All three sensors were tested in a maple tree, which is approximately 40 centimeters in diameter, over a period of five days near the end of August 2008. Data was collected on a Campbell Scientific CR-1000 data logger at a 1-s sampling interval. Sap flow rates and pulse velocities were calculated every 15 min. Based on the observed minimum sap flow rates, the 24-h period for determining the maximum temperature difference for the TDM calculations was set to start at 06:00:00. Corrections for the temperature and constant heat probes, as described in Section 3.1.5, were applied to both sets of self-made sap flow sensors. The correction equations for the TDM constant heat probe (HP) and reference temperature probe (TP) are:

(4) $${\mathrm{HP}}_{\mathrm{temp}}\;\left(\mathrm{corrected}\right)=1.1065\cdot {\mathrm{HP}}_{\mathrm{temp}}\;(\mathrm{raw})-1.4742$$

(5) $${\mathrm{TP}}_{\mathrm{temp}}\;\left(\mathrm{corrected}\right)=1.0539\cdot {\mathrm{TP}}_{\mathrm{temp}}\;\left(\mathrm{raw}\right)-2.8209$$

The correction equations for the HRM downstream (DP) and upstream (UP) temperature probes, respectively, are:

(6) $${\mathrm{DP}}_{\mathrm{temp}}\;\left(\mathrm{corrected}\right)=1.0532\cdot {\mathrm{DP}}_{\mathrm{temp}}\;\left(\mathrm{raw}\right)-2.6328$$

(7) $${\mathrm{UP}}_{\mathrm{temp}}\;\left(\mathrm{corrected}\right)=1.0451\cdot {\mathrm{UP}}_{\mathrm{temp}}\;\left(\mathrm{raw}\right)-2.5195$$

**Figure 8.**
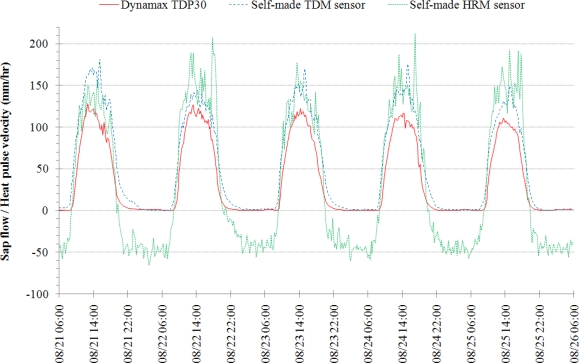
21–26 August 2008 comparison of Dynamax TDP30 sap flow sensor (solid red line) to self-made TDM (dashed blue line) and HRM (dotted green line) sensors in a maple tree.

The results of these measurements are shown in Figure 8. Due to the complexities in determining the dynamic *in situ* thermal properties of the tree’s woody matrix, only the heat pulse velocity for the HRM sensor is plotted.

Based on the general shape of the diurnal cycle and magnitude of the measurements (for the TDM), Figure 8 suggests that the self-made sensors are capable of adequately capturing daily sap flows. It can be seen that the self-made TDM sensor results in higher sap flow values compared to the Dynamax TDP30 sensor for all five days of measurements. The magnitude differences are not substantially different given that the measurements were made in different locations on the tree. Notice that the peak sap flow occurs near the same time of day on each of the five days for the self-made TDM sensor. The spike in sap flow for the self-made TDM sensor occurs between 15:00 and 15:30 each day. The commercial TDP30 sensor peaks sometime between 13:00 and 15:00 except for the first day where it peaks closer to 12:45. The morning and evening sap flow trends are similar in slope and time of occurrence for both the self-made TDM and commercial TDP30 sensors.

Figure 8 shows that the HRM sensor results are in good agreement with the self-made TDM sensor. The onset of positive sap flow in the HRM sensor occurs at the same time for both the self-made TDM and commercial TDP30 sensors. The high variability during times of peak sap flow rates makes it difficult to compare the time of peak sap flow occurrence. Note that the HRM calculations are for the heat pulse velocity, not the sap velocity, so the magnitudes of the HRM results cannot be directly compared to the results of the self-made TDM or commercial TDP30 sensors. While the self-made TDM and commercial TDP30 sensors show a lagging tail to the day’s sap flow measurements, the HRM sensor is quick to measure the end of the day’s positive sap flow and immediately begins to measure reverse sap flow rates. The negative sap flow rates in the HRM curve correspond to reverse sap flow (downward movement) that the HRM sensor design measures. The magnitude of the reverse rates are close to others reported using the HRM method with measurements showing reverse sap flow around 25 mm·h^−1^ for tap roots in *Eucalyptus camaldulensis* [21] and approximately 100 mm·h^−1^ in the main stem of *Eucalyptus salmonophloia* [22]. It is interesting to note that the magnitude of the nighttime HRM sap velocities is approximately one-third of the daytime rates.

**Figure 9.**
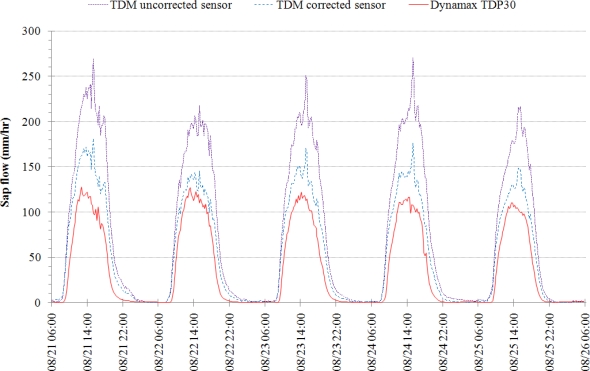
Comparison of sap flow calculations between the Dynamax TDP30 sensor (solid red line), temperature-corrected self-made TDM sensor (dashed blue line) and uncorrected self-made TDM senor (dotted purple line).

TDM sap flow calculations were also made based on the raw temperature measurements from the self-made sap flow probes. Due to the calculation method for the HRM, the pulse velocity calculations were not significantly affected by the uncorrected temperature data (maximum deviation was 1.16 mm·h^−1^). The TDM, however, showed a significant difference in sap flow velocities between the corrected and uncorrected temperature calculations. Deviations between the corrected and uncorrected temperature calculations were in excess of 90 mm·h^−1^ near peak hours, as shown in Figure 9.

## 5. Discussions and Conclusions

There are some important considerations that need to be noted when comparing the results of the self-made and commercial TDM sensors. The first consideration is with the method used for setting the constant heat input of both sensors. The heat input level of the self-made and commercial sensor was based in this research on matching the maximum temperature difference, Δ*T*_o_ in Equation (1), similar to that of [13] as described in [8]. Adjusting the heat input in this way may alter the heat field around the sensor probe such that Granier’s empirical equation used to estimate the sap flow may no longer be completely valid. There is yet to be a study that examines the effects of adjusting the heat field on TDM sensor measurements. The second concern is with the circumferential variations in sap flow. The measurements shown in Figure 8 were made simultaneously on the same tree. The two sap flow sensors were located approximately 90^o^ apart from one another and at slightly different elevations (approximately 12 cm separation). Numerous studies have shown and reported circumferential sap flow variations with fluctuations as high as 50% [5,13,23,24]. Lastly, there have been suspicions that the Dynamax TDP sap flow sensor systematically underestimates transpiration [8,25]. Currently there is no information regarding the calibration of the Dynamax TDP sap flow sensor with regards to Granier’s original empirical equation. These concerns may account for the apparent over-estimation in sap flow results given by the self-made sensors compared to their commercial counterparts. Considering these factors and the general shapes of the diurnal patterns (see Figure 8) of the self-made sap flow sensors, it is encouraging to see that the self-made TDM sensors can provide acceptable sap flow measurements compared to the commercial ones (Dynamax TDP30) with roughly only 2% of the cost of the commercial sensors.

It should be noted that calibrating Equation (1) for the TDM to specific tree species is important. The sensor design given in this paper does not correct nor address the validation of Equation (1) for individual trees. Therefore, the researcher must exercise caution when analyzing sap flow measurements made on tree species that have not been properly calibrated for the TDM.

The large fluctuations present in the peak sap flow values shown in Figure 8 for the self-made TDM and HRM and in the nighttime sap flow rates for the self-made HRM can be attributed to the method used to calculate these sap flow values. The 1-s dataset collected for the TDM measurements was sampled at every 15-min value. Given the variance in the measurement values, it is possible that the measurements made at the 15 min time stamps were not the best representative values for the calculations. If the sap flow was instead calculated at the 1-s time interval and averaged over each 15 min period, this would smooth the fluctuations observed in the self-made data. This suggests that the variance in the commercial sensor is much lower than that with the self-made sensors. The HRM calculation averages over 40 data points for each 15 min sap flow value, *i.e.*, measurements from 60–100 s. If either *v*_1_ or *v*_2_ are found to be negative or zero, then their ratio, *v*_1_/*v*_2_, is assumed zero. This was chosen to avoid holes in the dataset. Therefore, sensor fluctuation could cause zero values within the 40 data points which would affect the average for that period.

The costs of the self-made sensors are considerably small, although this may be compensated by the time and effort required to build and test them. One set of sap flow probes can be built on average in approximately one hour. This is based on moderate to large scale production where many sensors are made at the same time. This method of construction increases efficiency and improves the craftsmanship by repeating the same procedure over and over again. While the quality checks and output calibration add additional time to the procedure, it is important to identify and eliminate as many errors as early in the deployment as possible. Figure 9 shows the potential problems that improper temperature correction can cause when using the TDM. It may be assumed that all sap flow sensors made by the same person under the same conditions will behave similarly. This may reduce the need for testing every individual sensor probe’s measurement output. Therefore, for self-made sensor applications, it may be better to use the HRM which is less susceptible to individual probe variations if one does not have time to calibrate individual probes.

In summary, (1) the most cost-effective method for sap flow monitoring is a combination of pulsed and thermal dissipation methods; (2) HRM and TDM sap flow sensors can be constructed based on three probe designs; (3) utilizing common parts from wholesale vendors saves money for large productions; (4) time and effort building sap flow sensors provides a unique educational experience for undergraduate students; (5) field tests show that careful quality control of sensor output is more important for the TDM sensor than the HRM sensor; (6) compared to the Dynamax TDP30 sap flow sensor, both the HRM and TDM self-made sensors provide an acceptable alternative to commercial sensors at a fraction of the price.

## Figures and Tables

**Table 1.** Material quantities for a temperature probe, constant heat probe, heat pulse probe and voltage regulator.

**Item**	**Temp. Probe**	**Const. Heat Probe**	**Heat Pulse Probe**	**Voltage Regulator**
Pipette (10 μL)	1 ea.	1 ea.	1 ea.	—
Heat shrink tubing	2 cm	2 cm	2 cm	—
Stainless steel needle	1 ea.	1 ea.	1 ea.	—
Cyanoacrylate	0.015 mL	0.015 mL	0.005 mL	—
PTFE tape	5 cm	15 cm	5 cm	—
Chromel TC wire	5.5 cm	5.5 cm	—	—
Constantan TC wire	5.5 cm	5.5 cm	—	—
Type E TC extend wire	10 cm	10 cm	—	—
NiCr heater wire	—	50.8 cm	22.7 cm	—
Heater extend wire	—	12 cm	12 cm	—
Electrical tape	—	5 cm	5 cm	—
Resistor (240 Ω)	—	—	—	2 ea.
Capacitor (0.1 μF)	—	—	—	4 ea.
Voltage reg. (LM317)	—	—	—	2 ea.
Potentiometer	—	—	—	2 ea.
Dual pc board	—	—	—	0.5 ea.
Extend wire	—	—	—	20 cm

**Table 2.** Commercial vendor 2011 part numbers and pricing for sap flow probe and voltage regulator materials.

**Item**	**Vendor**	**Part No.**	**Price/unit**
Pipette (10 μL)	Cole-Parmer	EW-07950-20	$13.50/100 pc.
Heat shrink tubing	DigiKey	A014B-4-ND	$1.69/1.22 m
Capacitor (0.1 μF)	DigiKey	P984-ND	$0.26 ea.
Resistor (240 Ω)	DigiKey	240QBK-ND	$0.07 ea.
Voltage reg. (LM317)	DigiKey	LM317TFS-ND	$0.68 ea.
Potentiometer	DigiKey	CT2263-ND	$1.55 ea.
Stainless steel needle	McMaster-Carr	75165A75A	$14.41/50 pc.
NiCr heater wire	McMaster-Carr	8880K85	$25.01/487.68 m
Heater extend wire	McMaster-Carr	9697T1	$35.00/76.20 m
Extend wire	McMaster-Carr	7587K911	$6.56/30.48 m
Electrical tape	McMaster-Carr	76455A22	$4.33/20.12 m
Cyanoacrylate	McMaster-Carr	74555A44	$4.92/0.1 oz.
PTFE tape	McMaster-Carr	4591K12	$1.74/15.24 m
Chromel TC wire	Omega	TFCH-005-50	$16.00/15.24 m
Constantan TC wire	Omega	TFCC-005-50	$16.00/15.24 m
Type E TC extend wire	Omega	EXTT-E-20-50	$72.00/15.24 m
Dual pc board	RadioShack	276-148	$1.99 ea.
